# Use of HIV counseling and testing and family planning services among postpartum women in Kenya: a multicentre, non-randomised trial

**DOI:** 10.1186/s12905-015-0262-6

**Published:** 2015-11-13

**Authors:** James Kimani, Charlotte E Warren, Timothy Abuya, Charity Ndwiga, Susannah Mayhew, Anna Vassall, Richard Mutemwa, Ian Askew

**Affiliations:** Population Council, General Accident Insurance House, Ralph Bunche Road, P.O. Box 17643-00500, Nairobi, Kenya; London School of Hygiene & Tropical Medicine, Department of Global Health and Development, 15-17 Tavistock Place, London, WC1H 9SH UK; London School of Hygiene & Tropical Medicine, Department of Population Studies, Keppel Street, London, WC1E 7HT UK

**Keywords:** Postnatal care, Family planning, HIV testing and counselling, Integration, Kenya

## Abstract

**Background:**

Addressing the postnatal needs of new mothers is a neglected area of care throughout sub-Saharan Africa. The study compares the effectiveness of integrating HIV and family planning (FP) services into postnatal care (PNC) with stand-alone services on postpartum women’s use of HIV counseling and testing and FP services in public health facilities in Kenya.

**Methods:**

Data were derived from samples of women who had been assigned to intervention or comparison groups, had given birth within the previous 0–10 weeks and were receiving postnatal care, at baseline and 15 months later. Descriptive statistics describe the characteristics of the sample and multivariate logistic regression models assess the effect of the integrated model of care on use of provider-initiated testing and counseling (PITC) and FP services.

**Results:**

At the 15-month follow-up interviews, more women in the intervention than comparison sites used implants (15 % vs. 3 %; *p* < 0.001), while injectables were the most used short-term method by women in both sites. Women who wanted to wait until later to have children (OR = 1.3; *p* < 0.01; 95 % CI: 1.1–1.5), women with secondary education (OR = 1.2; *p* < 0.05; 95 % CI: 1.0–1.4), women aged 25–34 years (OR = 1.2; *p* < 0.01; 95 % CI: 1.1–1.4) and women from poor households (OR = 1.6; *p* < 0.001; 95 % CI: 1.4–1.9) were associated with FP use. Nearly half (47 %) and about one-third (30 %) of mothers in the intervention and comparison sites, respectively, were offered PITC. Significant predictors of uptake of PITC were seeking care in a health center/dispensary relative to a hospital, having a partner who has tested for HIV and being poor.

**Conclusions:**

An integrated delivery approach of postnatal services is beneficial in increasing the uptake of PITC and long-acting FP services among postpartum women. Also, interventions aimed at increasing male partners HIV testing have a positive effect on the uptake of PITC and should be encouraged.

**Trial registration:**

ClinicalTrials.gov NCT01694862

## Background

Addressing the postnatal needs of new mothers is a neglected area of care throughout sub-Saharan Africa. Data from Demographic and Health Surveys (DHS) in 30 developing countries revealed that, on average, only 28 %of women with non-institutional births received any form of postnatal care (PNC) [1].

Unintended pregnancies during the first 12 months following delivery and vertical transmission of HIV during labor and delivery are some of the challenges faced by women during the extended postpartum period. During the extended postpartum period many women want to delay or avoid pregnancies, but are not using a modern contraceptive method [2–6]; indeed, data from 27 Demographic and Health Surveys (DHS) found that 67 % of women who gave birth within the previous year had an unmet need for family planning (FP) [7]. Other studies have shown similar challenges faced by women during the extended postpartum period [8–11].

Evidence from recent studies has shown that substantial proportions of women living with HIV have an unmet need for FP during the postpartum period. Studies in Zambia and Kenya found that 39 and 65 %, respectively, of HIV-positive postpartum women reported that they had a regular sexual partner but were not using any FP method [12]. Moreover, use of prevention of mother-to-child HIV transmission (PMTCT) services does not necessarily influence postpartum use of contraceptive methods (except for condoms) [11]. Further, even if FP counseling and information is available in PMTCT programs to postpartum women living with HIV, this does not necessarily translate into their initiating contraception, despite the unmet need [11].

Few developing countries have mechanisms in place to ensure that mothers and their newborns are assessed early and monitored during the initial six-week period as recommended by WHO [13], which contributes to a discontinuity with services received during pregnancy and delivery and limits linkages to other key services for new mothers, including family planning, HIV testing and counseling, and HIV care for women and their infants living with HIV. Evidence has shown that providing a continuum of care from antenatal care (ANC), delivery, postnatal car (PNC) and beyond results in improved maternal and neonatal health outcomes [14–16]. For example, in Swaziland, integration of PMTCT into postnatal care led to considerable improvements in follow-up visits during the first 3 days postpartum, a significant increase in the proportion of postpartum women and their partners who got tested for HIV, increase in proportion of women and infants who received HIV treatment and care and significant improvements in the proportion of mothers practicing exclusive breastfeeding [14]. Studies in Kenya that evaluated models for integrating and strengthening HIV screening and management and FP into postnatal care for mothers and their newborns [17, 18] showed that integrated services were feasible and acceptable and contributed to significant improvements in uptake and quality of services.

HIV testing during antenatal provides an opportunity for HIV-infected women to access care and treatment to reduce the risk of mother-to-child HIV transmission (MTCT) and improve maternal health. For women who test negative during antenatal screening, they may feel that neither they nor their babies are at risk for HIV [19]. However, HIV may be acquired during pregnancy and postpartum period and would not be detected unless repeat HIV testing is conducted. Evidence has shown that incidence of HIV is high in the postpartum period and in turn increases the risk of vertical transmission of HIV [20]. Guidelines recommend provider-initiated HIV testing and counselling, including HIV testing and counselling for women of unknown status at labor and delivery, or postpartum during the breastfeeding period, and re-testing of women previously tested HIV-negative [21].

This study contributes to this limited evidence base by assessing the effect of integrating HIV PITC and FP services into PNC services on the use of PITC and FP services among new mothers attending postnatal care in public health facilities in Kenya. Specifically, the study aims to assess the effectiveness of integrated with stand-alone services on postpartum women’s use of HIV counseling and testing and FP services.

## Methods

### Description of intervention

The Integra Initiative was a multi-country research study measuring the feasibility, effects and costs of integrated HIV and sexual and reproductive health services in Kenya and Swaziland. The integrated HIV and PNC service model developed explicit linkages with FP services and relevant HIV/AIDS services, for the mother and her baby. The intervention focused on strengthening existing postpartum consultations during pre-discharge, 1 week, and six-week, additional consultations were introduced at 6 months to enable women to access time-relevant services for themselves and their babies. Moreover, information about and encouragement to receive this full package of postpartum services was made during antenatal-care consultations to increase continuum of care of essential services. The services included repeat HIV testing for mother, HIV testing for infant and referral to HIV services for HIV positive mothers and infants, as well as referrals for clients requiring additional services. Before recruitment of participants, providers in study intervention facilities were trained in provision of integrated services using a Balanced Counselling Strategy Plus algorithm (BCS+) and a standardised mentorship strategy described elsewhere [22]. Study implementation begun after intervention-facility providers were certified as attaining a pre-determined minimum level of clinical skills. The study methodology used to evaluate the intervention is described in detail elsewhere [23].

The Integra Initiative is a registered trial. The trial is registered at http://www.ClinicalTrials.gov, number NCT01694862.

### Study population and sampling

The data used in this paper were collected through the INTEGRA Initiative. The study sample was selected from women aged 15 to 49 years, who had given birth within the previous 0–10 weeks and were receiving postnatal care. The respondents were recruited between February 2010 and May 2010 from public health facilities (in peri-urban and rural areas of Eastern Province in Kenya) as part of a prospective cohort study that aimed at measuring several reproductive health indicators, including fertility intentions, pregnancy (planned and unintended), use of FP, HIV status and other sexual and reproductive behaviors. A total of 1693 women (815 intervention and 878 comparison) were recruited at baseline. At 15-month follow-up, 573 and 631 women in the intervention and comparison groups, respectively, were followed up and interviewed. For this paper, the main outcomes of interest were changes in uptake of PITC and FP services. Participant allocation to either intervention or control arm occurred through a pre-recruitment treatment allocation algorithm for health facilities.

The health facilities were purposively selected based on the range of services provided (family planning, voluntary counseling and testing (VCT), PMTCT, postnatal care and immunizations); high client load (>50 infants per month receiving their first immunizations at 6 weeks at the PNC-HIV clinics); a minimum of two providers qualified in and currently delivering FP services; and no current provision of fully integrated PNC and HIV services. To maximize equivalence between the intervention and comparison facilities, a pair-wise matching sampling design was used to assign the facilities into the two groups. Randomization of the sites was not done because of a number of considerations. First, in discussion with the MOH, the project team was advised which districts to select for project implementation. Second, the need to build on work that had been done in the same sites by previous studies informed the decision to implement the Integra Initiative in those sites [24, 17]. Details about the study population, sampling strategy, sample size calculations, selection of study sites and indicators are published elsewhere [25].

### Data collection

A closed-ended questionnaire was used to collect data on a range of factors, including the women’s use of FP, whether they ever had an HIV test, whether the provider offered HIV counseling and testing at current facility visit, other sexual and reproductive health (SRH) behaviors and health-seeking behaviors. Teams of trained research assistants conducted the interviews using hand-held personal digital assistants (PDAs) loaded with the questionnaire tool that had been translated into Swahili.

### Ethical considerations

The conduct of the INTEGRA Initiative was approved by the Kenya Medical Research Institute (KEMRI) Ethical Review Board (IRB approval numbers 113 and 114), Population Council’s Institutional Review Board (IRB approval numbers 443 and 444) and the Ethics Review Committee of the London School of Hygiene & Tropical Medicine (LSHTM) (IRB approval number 5426). The research assistants were trained in research ethics and obtained informed consent from all respondents.

Every respondent was given full study information and gave their informed consent prior to being interviewed. Participants indicated their consent by signature or witnessed thumbprint. For study participants who were younger than 18 years of age, informed consent was also sought. Rather than asking their assent as children with an associated parental permission, parenting adolescents between the ages of 15–17 years were considered ‘emancipated minors’ due to their pregnancy status. There are several national policies and guidelines that advocate for the autonomy of adolescents with respect to their reproductive healthcare and rights. The National Guidelines for HIV Testing and Counselling [26] references emancipated youth and adolescents and notes that “children may be tested with the consent of a parent or guardian, or may give their own if they are symptomatic, pregnant, married, a parent, or engaged in behavior that puts them at risk of contracting HIV,” and the Kenya National Guidelines for Research and AIDS Vaccines/AIDS Vaccines [27] states that “for mature minors (<18 years but married, pregnant, a parent or engaging in behavior that puts them at risk for STI/HIV infection), consent should be obtained from the minor”. The National Reproductive Health Research Guidelines details that “unless specific legal provisions exist, consent to participate in research should be given only by the adolescents” [28].

### Data analysis

Data analysis used the complete-case analysis and focused only on the cases that were interviewed at 15-month follow-up (573 and 631 women in the intervention and comparison groups, respectively). Analyses were performed on data collected at baseline and 15-month follow-up using Stata® version ten. Descriptive statistics were used to describe the characteristics of the sample. Separate multivariable logistic regression models were used to assess the effect of the PNC and HIV integration model on uptake of PITC and FP services. The two primary dependent variables were i) PITC offered, and ii) use of a modern family planning method, both at 15-month follow-up.

The key independent variable was exposure to the intervention activities (denoted as study group) and was dichotomized into two categories (1 = intervention and 0 = comparison). Other covariates that were identified in previous research ([1, 25, 29, 30]) and considered in the analyses included facility type (hospital and health center/dispensary), whether partner had been tested for HIV, desire to have more children, number of children desired, whether a woman wanted to be pregnant with last pregnancy, education level, age of respondents, marital status and household socioeconomic status.

For the bivariate analysis, Chi-square test (*X*^2^) and Fisher’s Exact test (where cell sizes were small (<5)) were used to test the association between the dependent variables and independent variables. To assess and correct for selection bias in the two study groups, propensity score analysis was used to balance the data and ensure improved precision in estimation of the treatment effect. Propensity scores were generated using logistic regression of the treatment (study group) variable on four covariates (education level, household socioeconomic status, facility type and whether a woman wanted to be pregnant with last pregnancy) that were significantly associated with the study group variable at baseline. The propensity score variable was grouped into quintiles for each contrast investigated, using the four covariates. Data-balance correction was checked and confirmed in each quintile on all covariates used in each propensity score model. Two types of models were fitted in the analysis. Firstly, two causal models were fitted in which the propensity score covariate was included to replace confounders, to determine the causal effect of the Integra Intervention on each of the two primary outcomes, over the study period. Secondly, two separate predictive models for PITC and FP were fitted, using the covariates earlier found to be strongly associated with the respective primary outcomes in univariable association tests. In the multivariable analysis, regression models also accounted for clustering effects at the facility level adapting methods used in previous studies [31, 32] and the results were presented as adjusted odds ratios. The threshold for significant results was set at *p* < 0.05 or above. The models also included an interaction term between the study group and study round (coded as 1 = 15-month follow-up and 0 = baseline) to assess the difference in the changes in uptake of PITC and FP services between intervention and comparison sites over time (that is, the difference-in-differences estimate).

## Results

### Descriptive analysis

Table 1 summarizes the study population characteristics by study group at baseline and 15-month follow-up. Overall, a similar proportion of women in both the intervention and comparison groups reported using family planning at 15-month follow-up. More women in the intervention than comparison sites used implants (15 % vs. 3 %; *p* < 0.001), while injectables were the most used short-term method by women in both sites (results not shown). About 47 % of respondents in the intervention sites were offered provider-initiated HIV testing and counseling (PITC), while 30 % of women in the comparison sites were offered PITC at 15-month follow-up. Majority of women in both study sites visited hospitals, reported being HIV-negative and visited a health facility for ante-natal services. Over 60 % of women in the intervention sites and about 55 % of women in the comparison sites reported they wanted to be pregnant with the last pregnancy. It is important to note due to attrition, the number of participants in each arm reduced between baseline and 15-month follow-up.

**Table 1:** Study population characteristics by study group

	Intervention		Comparison	
Variable	Baseline (*N* = 815)	15-month follow-up (*N* = 573)	Baseline (*N* = 878)	15-month follow-up (*N* = 631)
	N (%)	N (%)	N (%)	N (%)
Used family planning				
No	624 (76. 6)	137 (23.9)	732 (83.4)	146 (23.2)
Yes	191 (23. 4)	436 (76.1)	146 (16.6)	484 (76.8)
Offered PITC				
No	690 (84.7)	180 (53.4)	791 (90.1)	248 (70.4)
Yes	125 (15.3)	157 (46.6)	87 (9.9)	104 (29.6)
Facility type				
Hospital	528 (64.8)	362 (63.2)	632 (72.0)	434 (68.8)
Health center/dispensary	287 (35.2)	211 (36.8)	246 (28.0)	197 (31.2)
Respondent’s HIV status				
HIV-negative	773 (96.6)	532 (97.6)	815 (96.3)	579 (95.2)
HIV-positive	27 (3.4)	13 (2.4)	31 (3.7)	29 (4.8)
Partner tested for HIV				
No	298 (42.0)	267 (59.5)	293 (39.9)	285 (55.7)
Yes	411 (58.0)	182 (40.5)	442 (60.1)	227 (44.3)
Went to clinic for ANC services at last pregnancy				
No	13 (1.6)	16 (2.8)	26 (3.0)	25 (4.0)
Yes	802 (98.4)	557 (97.2)	852 (97.0)	606 (96.0)
Whether wanted to be pregnant with last pregnancy				
Wanted to wait until later	250 (30. 7)	166 (29.0)	261 (29.7)	188 (29.8)
Wanted to be pregnant	477 (58.5)	353 (61.6)	488 (55.6)	344 (54.5)
Didn’t want to have any more children	88 (10.8)	54 (9.4)	129 (14.7)	99 (15.7)
Desires more children				
No	405 (49.7)	298 (52.3)	478 (54.4)	346 (55.1)
Yes	329 (40.4)	262 (46.0)	321 (36.6)	257 (40.9)
Undecided	81 (9.9)	10 (1.7)	79 (9.0)	25 (4.0)
Average number of children desired (standard deviation)	3.1 (1.3)	3.5 (1.3)	3.0 (1.3)	3.3 (1.2)
Education				
Primary and below	559 (68.6)	365 (63.7)	547 (62.3)	402 (63.7)
Secondary	174 (21.3)	142 (24. 8)	252 (28.7)	176 (27.9)
College	82 (10.1)	66 (11.5)	79 (9.0)	53 (8.4)
Age of respondents				
15-24 years	424 (52.0)	300 (52.4)	441 (50.2)	330 (52.3)
25–34 years	316 (38.8)	227 (39.6)	358 (40.8)	252 (39.9)
35+ years	75 (9.2)	46 (8.0)	79 (9.0)	49 (7.8)
Marital status				
Single	130 (15.9)	94 (16.4)	148 (16.9)	100 (15.8)
Married	659 (80.9)	457 (79.8)	707 (80.5)	512 (81.1)
Divorced/separated/widowed	26 (3.2)	22 (3.8)	23 (2.6)	19 (3.01)
Household SES				
Poor	394 (48.3)	335 (58.5)	283 (32.2)	342 (54.2)
Non-poor	421 (51.7)	238 (41.5)	595 (67.8)	289 (45.8)

#### Uptake of provider-initiated HIV testing and counseling (PITC)

Table 2 describes the association between study sample characteristics and provider-initiated HIV testing and counseling by study group at 15-month follow-up. The results show that in the intervention sites, nearly half of the women who visited hospitals compared to health centers/dispensaries (49 % vs. 41 %; *p* < 0.166) were offered PITC. Similarly, in the comparison sites, women who went to hospitals relative to health centers/dispensaries were more likely to be offered PITC; however, the differences were not statistically significant. In both study sites, having a partner who has been tested for HIV was significantly associated with being offered PITC. Being offered PITC did not differ significantly by education, age, marital status and household socioeconomic status in the two study sites.

**Table 2:** Study population characteristics and association with provider-initiated HIV testing and counseling (PITC) at 15-month follow-up

	Offered PITC			
Variable	Intervention		Comparison	
	N (%)	*p*-values	N (%)	*p*-values
Facility type				
Hospital	118 (48.9)	0.166	82 (31.3)	0.219
Health center/dispensary	39 (40.6)		22 (24.4)	
Respondent’s HIV status				
HIV-negative	157 (48.6)	*	97 (30.4)	0.629
HIV-positive	0 (0.0)		5 (23.8)	
Partner tested for HIV				
No	54(36.2)	***	38 (22.0)	**
Yes	74 (60.2)		52 (38.0)	
Went to clinic for ANC services at last pregnancy				
No	2 (33.3)	0.689	4 (36.4)	0.738
Yes	155 (46.8)		100 (29.3)	
Education				
Primary and below	94 (44.6)	0.528	64 (28.1)	0.705
Secondary	45 (51.7)		30 (31.9)	
College	18 (46.2)		10 (33.3)	
Age of respondents				
15–24 years	88 (48.6)	0.674	55 (30.4)	0.791
25–34 years	58 (43.6)		42 (29.6)	
35+ years	11 (47.8)		7 (24.1)	
Marital status				
Single	21 (42.0)	0.696	15 (32.6)	0.880
Married	129 (47.1)		86 (29.2)	
Divorced/separated/widowed	7 (53.9)		3 (27.3)	
Household SES				
Poor	96 (48.0)	0.530	58 (29.4)	0.962
Non-poor	61 (44.5)		46 (29.9)	

*p < 0.05; **p < 0.01; ***p < 0.001; *X* ^2^test and Fisher’s Exact test were used to test the association between provider-initiated HIV testing and counseling and explanatory variables

#### Uptake of family planning (FP)

Results on uptake of FP at 15-month follow-up are presented in Table 3. Type of facility was significantly associated with uptake of FP. In the intervention sites, a significantly higher proportion of women who went to hospitals compared to health centers/dispensaries (81 % vs. 68 %; *p* < 0.001) reported using FP. In the comparison sites, a slightly higher proportion of women who visited a hospital reported using FP compared to women who went to a health center/dispensary, however, these differences were not statistically significant. Further analysis (results not shown) showed that injectables were the most used short-term method in both sites, however, more women in the intervention than comparison sites reported using implants (15 % vs. 3 %; *p* < 0.001).

**Table 3:** Study population characteristics and association with uptake of family planning at 15-month follow-up

	Uptake of family planning			
Variable	Intervention		Comparison	
	N (%)	*p*-values	N (%)	*p*-values
Facility type				
Hospital	292 (80.7)	***	340 (78.5)	0.135
Health center/dispensary	144 (68.3)		144 (73.1)	
Desires more children				
No	231 (77.5)	0.130	261 (75.4)	0.722
Yes	198 (75.6)		200 (78.1)	
Undecided	5 (50.0)		20 (80.0)	
Number of children desired^a^				
3 and below	263 (78.5)	0.120	294 (77.0)	0.903
4 and above	172 (72.9)		186 (76.5)	
Whether wanted to be pregnant with last pregnancy				
Wanted to be pregnant	273 (77.3)	0.519	269 (78.4)	0.475
Wanted to wait until later	121 (72.9)		143 (76.1)	
Didn’t want to have any more children	42 (77.8)		72 (72.7)	
Education				
Primary and below	274 (75.1)	0.664	312 (77.6)	0.768
Secondary	112 (78.9)		131 (74.9)	
College	50 (75.8)		41 (77.4)	
Age of respondents				
15–24 years	232 (77.3)	0.668	252 (76.4)	0.908
25–34 years	171 (75.3)		195 (77.7)	
35+ years	33 (71.7)		37 (75.5)	
Marital status				
Single	68 (72.3)	0.582	72 (72.0)	0.142
Married	352 (77.0)		400 (78.3)	
Divorced/separated/widowed	16 (72.7)		12 (63.2)	
Household SES				
Poor	262 (78.2)	0.158	267 (78.1)	0.420
Non-poor	174 (73.1)		217 (75.4)	

***p < 0.001; *X* ^2^test was used to test the association between use of a family planning method and explanatory variables; ^a^categories based on the median number of children (median = 3)

### Multivariate analysis

#### Effect of the intervention on the uptake of provider-initiated HIV testing and counseling (PITC) and family planning (FP) services

As presented in Table 4, the odds in the uptake of PITC services were higher the in intervention sites compared to the comparison sites (OR = 1.6; *p* < 0.01; 95 % CI: 1.2–2.2). The difference in the changes in uptake of PITC between intervention and comparison sites at baseline and 15-month follow-up (i.e., the difference-in-differences estimate, as denoted by the interaction term between study group and study round) was not statistically significant. This means that the increase in the proportion of women who were offered PITC in the intervention sites was not significantly different from the change observed in the comparison sites.

**Table 4:** Adjusted odds ratios (AORs) and 95 % confidence intervals (CIs) for effect of intervention on uptake of provider-initiated testing and counseling and FP services

	Offered PITC	
Variables	Adjusted OR^a^	95 % CI
Study group (Ref = Comparison)		
Intervention	1.6**	1.2–2.2
Study round(Ref = Baseline)		
15-month follow-up	3.7***	2.7–5.2
Study group × study round^a^	1.3	0.8–2.0
	Uptake of FP	
	Adjusted OR^a^	95 % CI
Study group (Ref = Comparison)		
Intervention	1.6***	1.3–2.0
Study round(Ref = Baseline)		
15-month follow-up	17.3***	13.3–22.5
Study group × study round^b^	0.6**	0.4–0.8

**p < 0.01; ***p < 0.001; *Ref* reference category; ^a^the following variables were included in the models: education level, household socioeconomic status, facility type and whether a woman wanted to be pregnant with last pregnancy; ^b^the difference in the changes in outcomes between intervention and comparison sites over time (i.e., the difference-in-differences estimate)

**Table 5:** Adjusted odds ratios (AORs) and 95 % confidence intervals (CIs) for predictors of provider-initiated testing and counseling

	Offered PITC	
Variables	Adjusted OR	95 % CI
Facility type (Ref = Hospital)		
Health center/dispensary	2.0***	1.6–2.5
Partner tested for HIV (Ref = No)		
Yes	1.4**	1.1–1.8
Went to clinic for ANC services at last pregnancy (Ref = No)		
Yes	0.4*	0.2–0.9
Respondent’s HIV status (Ref = HIV negative)		
HIV-positive	0.7	0.4–1.4
Education (Ref = Primary and below)		
Secondary	1.1	0.9–1.5
College	1.2	0.8–1.9
Age of respondents (Ref = 15–24 years)		
25–34 years	1.0	0.8–1.3
35+ years	1.4	1.0–2.0
Marital status (Ref = Married)		
Single	0.8	0.6–1.2
Divorced/separated/widowed	1.4	0.7–2.8
Household SES (Ref = Non-poor)		
Poor	1.3*	1.0–1.6

*p < 0.05; **p < 0.01; ***p < 0.001 *Ref* reference category

**Table 6:** Adjusted odds ratios (AORs) and 95 % confidence intervals (CIs) for predictors of uptake of family planning

	Uptake of FP	
Variables	Adjusted OR	95 % CI
Facility type (Ref = Hospital)		
Health center/dispensary	0.7***	0.6–0.9
Whether wanted to be pregnant with last pregnancy (Ref = Wanted to be pregnant)		
Wanted to wait until later	1.3**	1.1–1.5
Didn’t want to have any more children	1.2	1.0–1.6
Desires more children (Ref = No)		
Yes	0.8**	0.7–1.0
Undecided	0.4***	0.3–0.5
Number of children desired	1.1***	1.1–1.2
Education (Ref = Primary and below)		
Secondary	1.2*	1.0–1.4
College	1.2	1.0–1.5
Age of respondents (Ref = 15–24 years)		
25–34 years	1.2**	1.1–1.4
35+ years	0.9	0.7–1.2
Marital status (Ref = Married)		
Single	0.6***	0.5–0.8
Divorced/separated/widowed	0.9	0.6–1.3
Household SES (Ref = Non-poor)		
Poor	1.6***	1.4–1.9

*p < 0.05; **p < 0.01; ***p < 0.001 *Ref* reference category

The results from the multivariable logistic models on uptake of family planning are also presented in Table 4. The likelihood of FP use was higher in the intervention sites compared to the comparison sites (OR = 1.6; *p* < 0.001; 95 % CI: 1.3–2.0). Based on the difference-in-differences estimate (interaction term between study group and study round), the increase in uptake of FP in the intervention sites was significantly lower (OR = 0.6; *p* < 0.01; 95 % CI: 0.4–0.8) compared to the comparison sites.

#### Factors associated with uptake of provider-initiated HIV testing and counseling (PITC) during the postnatal period

Women who visited health centers/dispensaries were twice more likely to be offered PITC (OR = 2.0; *p* < 0.001; 95 % CI: 1.6–2.5) compared to women who went to hospitals. Having a partner who has tested for HIV was significantly was associated with being offered PITC (OR = 1.4; *p* < 0.01; 95 % CI: 1.1–1.8) relative to women whose partners had not tested for HIV. Women who reported going to a health facility for ante-natal services were less likely to be offered PITC (OR = 0.4; *p* < 0.05; 95 % CI: 0.2–0.9) (Table 5).

#### Factors associated with uptake of family planning (FP) during the postnatal period

Women who went to health facilities/dispensaries had significantly lower odds of using FP (OR = 0.7; *p* < 0.001; 95 % CI: 0.6–0.9) compared to women who went to hospitals. Women who wanted to wait until later were significantly associated with uptake of FP (OR = 1.3; *p* < 0.01; 95 % CI: 1.1–1.5).to get pregnant compared to women who wanted to be pregnant with last pregnancy. Women who desired more children and those who were undecided had lower odds of using FP. The odds of using FP increased as women attained the number of children desired (OR = 1.1; *p* < 0.001; 95 % CI: 1.1–1.2). Education and age were also significant determinants of uptake of FP. Women with secondary education were 1.2 times (*p* < 0.05; 95 % CI: 1.0–1.4) more likely to use FP compared to women with primary education or lower. Women aged 25–34 years had a higher likelihood of using FP compared to women aged 15–24 years (OR = 1.2; *p* < 0.01; 95 % CI: 1.0–1.4). Marital status was also an important predictor of uptake of family planning. Specifically, single women were significantly less likely to use a FP method (OR = 0.6; *p* < 0.001; 95 % CI: 0.5–0.8) compared to married women. Women from poor households were 1.6 times (*p* < 0.001; 95 % CI: 1.4–1.9) more likely to use FP compared to women from non-poor households (Table 6).

## Discussion

The objective of this paper was to assess the effect of integrating HIV and postnatal care services on the uptake of provider-initiated HIV testing and counseling and family planning services among women attending postnatal care in public health facilities in Kenya. The findings show there was improvement in the uptake of PITC in the sites that implemented an integrated approach in the delivery of postnatal and HIV services compared to comparison sites. Previous studies also found that integrating postnatal and HIV services had a positive effect on uptake of HIV counseling and testing, particularly PITC [14].

With regard to type of health facility, health centers/dispensaries were associated with a higher likelihood of offering PITC to mothers compared to hospitals. Our analysis showed that women who attended antenatal clinics (ANC) were less likely to be offered PITC. This suggests that the women may have been offered HIV counseling and testing and, therefore, there was no need for them to be tested again during the PNC visit. Similar to previous research [29, 30], having a partner who has been tested for HIV was also an important predictor of being tested for HIV. One plausible explanation for this finding is that having a partner who has been tested for HIV increases the level of awareness about HIV/AIDS and serves as a motivating factor to get tested. Women aged 35 years and above were associated with greater odds of being offered PITC. This observation is consistent with past research on determinants of pathways to HIV testing in Kenya [25]. This may suggest an assumption by providers that the older a person is the more likely they have been exposed to the risk of HIV infection. Women from poor households were also associated with a higher likelihood of being offered PITC compared to their counterparts from non-poor households.

We observed that uptake of long-term FP methods, specifically implants, was higher among women in the intervention sites compared to their counterparts in the comparison sites. This is an important finding which shows that more women used appropriate long-term methods rather than less reliable short-term methods. The intervention involved the implementation of a number of activities including promotion of long-acting reversible contraception (LARC), ensuring availability of minimum levels of supplies for integrated services including FP commodities and improving provider capacity in offering LARC. In the multivariable analyses (difference-in-difference results), however, we observed that the uptake of FP in the intervention sites was significantly lower compared to comparison sites. Presumably, this would partly be explained by the higher FP use in the intervention sites compared to the comparison sites and, therefore, where there are more users we would expect fewer new users of FP services.

We found that a number of factors were significant predictors of uptake of FP, including type of health facility, whether a woman wanted to be pregnant with last pregnancy, whether a woman desires more children, number of children desired, education level, age, marital status and household socioeconomic status. Women seeking PNC services in lower level facilities (health centers/dispensaries) were associated with a lower likelihood of using a FP method compared to hospitals. One probable reason for this observation is the issue of stock-out of essential FP commodities and supplies, which might affect lower level facilities more than hospitals. Frequent stock-outs have been cited by previous studies as one of the barriers to effective provision of integrated services [16, 33]. This finding has key implications with regard to reducing the risk of unwanted pregnancies among women visiting lower-level facilities for postnatal consultations. Women who wanted to wait until later to have a child had a higher likelihood of using FP compared to those who wanted to be pregnant. This is an encouraging outcome which indicates that women who wanted to delay or space child birth were taking necessary steps to achieve this goal. Number of children desired was an important predictor of FP use. As the number of children desired increased, women were more likely to use a FP method. This suggests that as women achieve the desired number of children they are motivated to adopt relevant family planning practices. Age was also associated with uptake of FP. Women aged 25–34 years were more likely to use a FP method compared to women aged 15–24 years. This suggests that more efforts are needed to encourage younger women to use family planning services. Marital status and household socioeconomic status were also important predictors of uptake of FP. Single women had a lower probability of using a FP method compared to married women. Women from poor households were associated with a higher likelihood of FP use compared to women from non-poor households. We could not find a plausible explanation for this observation and future research needs to investigate this outcome.

The findings from our study have important policy implications. Our study highlights the need by the government to address gaps in the provision of essential FP commodities and supplies, particularly in lower level health facilities. The results showed that women who visited these facilities were less likely to receive a FP method during their postnatal consultations relative to women who went to hospitals. It is imperative that these gaps are addressed to ensure that women’s sexual and reproductive health needs are met, including helping the women achieve their fertility intentions and reducing the risk of unwanted pregnancies.

The lost-to-follow-up (LFU) was 30 and 28 % for the intervention and comparison groups, respectively. The LFU was within the anticipated range (30 %) and a sensitivity check suggested that it was random not systematic, and, therefore, no effect on the findings.

### Limitations

Our study had a number of limitations that need to be highlighted. First, the Integra Initiative from which data for this study were derived was implemented in a ‘real world setting’, which means that it was difficult to control for the wide variety of factors that were likely to impact on the intervention, including contamination of comparison sites. For example, at the time when Integra was being implemented, the Government of Kenya initiated a policy change by rolling out the National Reproductive Health and HIV/AIDS Integration Strategy [34], which aimed at restructuring and reorienting health systems to provide sustainable integrated reproductive health and HIV/AIDS services. It is possible that facilities in the comparison sites may have ‘integrated’ independent of Integra inputs, or challenges in implementation may have hindered integration in the intervention sites. Future studies need to address these limitations by utilizing an integration index to measure the precise degree of integration at facility level and the effect on health outcomes. Third, there was no randomization of facilities to intervention or control arms of the study. To mitigate the limitation of non-randomization, we applied propensity score analysis to each fitted causal model by adapting methods to correct for selection bias in the data due to non-randomized design of the Integra Initiative [35, 36]. Finally, our findings are limited to Kenya and cannot be generalized to other African countries.

## Conclusion

In conclusion, our study findings point to the potential success of an integrated delivery approach of postnatal services in increasing the uptake of long-acting FP methods and PITC services among women attending PNC clinics. Interventions aimed at increasing male partners HIV testing should be encouraged since uptake of PITC was positively associated with having a partner who has tested for HIV.
